# Ultrafast Synthesis of Calcium Vanadate for Superior Aqueous Calcium-Ion Battery

**DOI:** 10.34133/2019/6585686

**Published:** 2019-11-29

**Authors:** Liyuan Liu, Yih-Chyng Wu, Patrick Rozier, Pierre-Louis Taberna, Patrice Simon

**Affiliations:** ^1^CIRIMAT, UMR CNRS 5085, Université Paul Sabatier Toulouse III, 118 route de Narbonne, 31062 Toulouse, France; ^2^RS2E, Réseau Français sur le Stockage Electrochimique de l'Energie, FR CNRS 3459, 80039 Amiens CEDEX, France

## Abstract

Recently, multivalent aqueous calcium-ion batteries (CIBs) have attracted considerable attention as a possible alternative to Li-ion batteries. However, traditional Ca-ion storage materials show either limited rate capabilities and poor cycle life or insufficient specific capacity. Here, we tackle these limitations by exploring materials having a large interlayer distance to achieve decent specific capacities and one-dimensional architecture with adequate Ca-ion passages that enable rapid reversible (de)intercalation processes. In this work, we report the high-yield, rapid, and low-cost synthesis of 1D metal oxides *M*V_3_O_8_ (*M* = Li, K), CaV_2_O_6_, and CaV_6_O_16_·7H_2_O (CVO) via a molten salt method. Firstly, using 1D CVO as electrode materials, we show high capacity 205 mA h g^−1^, long cycle life (>97% capacity retention after 200 cycles at 3.0 C), and high-rate performance (117 mA h g^−1^ at 12 C) for Ca-ion (de)intercalation. This work represents a step forward for the development of the molten salt method to synthesize nanomaterials and to help pave the way for the future growth of Ca-ion batteries.

## 1. Introduction

To satisfy the demand for portable electronics and the electric vehicle market, the development of rechargeable battery technique plays a significant role. In the past decades, considerable efforts have been devoted to monovalent ion batteries such as Li^+^, Na^+^, and K^+^ intercalation materials [1–3]. Recently, researchers have started to pay more attention to multivalent charge carriers [4–9]. Among them, various divalent metal ions have been proposed as alternatives to monovalent ions, including Ca^2+^ [4], Mg^2+^ [5], or Zn^2+^ [6]. The adoption of divalent ions results in a potential improvement in the specific capacity and hence a significant boost in the energy density of cells compared to monovalent ion cells (2F (1F = 96500C mol^−1^) exchanged per mole of divalent metal ion intercalated instead of 1F for monovalent, for instance). Furthermore, the abundance of these divalent elements largely reduces the cost of storage systems and relieves the stress of the lack of lithium resources [4].

Nevertheless, multivalent-ion electrodes suffer from slow cation diffusion because of the strong binding between the multivalent ions and the negatively charged active materials [4, 5]. Thanks to the low charge density of Ca-ion (see [Supplementary-material supplementary-material-1]), this problem has been effectively alleviated in Ca-ion intercalation electrodes compared to the other multivalent-ion materials [4, 5]. Besides, the standard reduction potential of Ca/Ca^2+^ (-2.9 V vs. normal hydrogen electrode, NHE) is 0.5 V lower than Mg^2+^/Mg (-2.4 V vs. NHE), 1.2 V lower than Al^3+^/Al (-1.6 V vs. NHE), and only 0.1 V higher than Li^+^/Li (-3.0 V vs. NHE) [4, 5, 7]. According to Gummow et al., the theoretical volumetric capacity of Ca^2+^ (2073 mA h ml^−1^) is higher than Na^+^ (1128 mA h ml^−1^) and K^+^ (591 mA h ml^−1^), similar to Li^+^ (2062 mA h ml^−1^) [8]. As reported by Monti et al., the hypothetical energy density of a graphite/Ca_3_Co_2_O_6_ Ca-ion battery (120 Wh l^−1^ and 50 Wh kg^−1^) is higher than both LFP/graphite and NVPF/HC configurations [10].

However, research activities on Ca-metal battery are still at preliminary stages due to the incompatibility between the metal Ca anode and the conventional organic electrolyte [9]: Ca plating reaction is difficult to achieve at room temperature in organic Ca-ion batteries since Ca^2+^ is difficult to transport through the solid electrolyte interphase film when Ca metal is used as an anode [11]. This problem remained unsolved until very recently when Wang et al. used Ca_7_Sn_6_ alloy to replace Ca metal as anode, but the cycling stability and coulombic efficiency are still waiting to be improved [11]. Differently from organic Ca-metal batteries, there is more success on the development of Ca-ion batteries where host materials such as organic polyimide can be used as the anode [4]; also, several materials such as K_0.31_MnO_2_·0.25H_2_O [12], Prussian blue analog copper hexacyanoferrate [13], or CaCo_2_O_4_ [14] have been proposed as cathodes for reversible Ca^2+^ (de)intercalation for aqueous Ca-ion batteries. We believe that the development of Ca-ion batteries would play a critical role in pushing the Ca-metal batteries forward.

Recently, Zn-ion batteries have won a lot of attention [15]. Among the cathode materials, many vanadium-based oxides have been reported such as Zn_0.25_V_2_O_5_·2H_2_O [16], Fe_5_V_15_O_39_(OH)_9_·9H_2_O [17], Zn_3_V_2_O_7_(OH)_2_·2H_2_O [6], H_2_V_3_O_8_ [18], LiV_3_O_8_ [19], and Na_2_V_6_O_16_·1.63H_2_O nanoribbon [20]. These vanadium-based oxides show excellent electrochemical performance in Zn-ion batteries thanks to their open-framework crystal structure and the existence of multiple oxidation states of vanadium. So far, no vanadate has been reported in aqueous Ca-ion batteries, which indicates that there might be a lot of interesting vanadium-based electrode materials remaining to be discovered for Ca-ion batteries. For organic Ca-ion batteries, only V_2_O_5_ has been reported as the cathode material, but the reversible capacity drops down to only 20% after only 4 cycles [21, 22]. Among the vanadium-based oxides, metahewettite-type CaV_6_O_16_·3H_2_O has been reported as a possible cathode material for Li-ion [23] and Zn-ion batteries [24]. It exhibits a layered structure composed by the stacking of V_6_O_16_ layers linked together by Ca^2+^ and H_2_O, which lying in the interlayer spaces, lead to a large interlayer distance which favors the intercalation of ions [24].

In this work, 1-dimensional CaV_6_O_16_·7H_2_O (CVO) samples were successfully prepared by using an ultrafast (synthesis time of only a few minutes) and high-yield (several grams can be easily prepared in one pot at lab scale) molten salt method (MSM). This compound was then characterized to assess its use as an electrode material in an aqueous Ca-ion battery. Interestingly, the MSM synthesis method can be extended to the preparation of a large variety (Li^+^ and K^+^ besides Ca^2+^) of ion intercalated vanadium oxides all with large interlayer distance, making this method promising to prepare nanomaterials for energy storage applications.

## 2. Results and Discussion

### 2.1. Material Characterization

**Figure 1:**
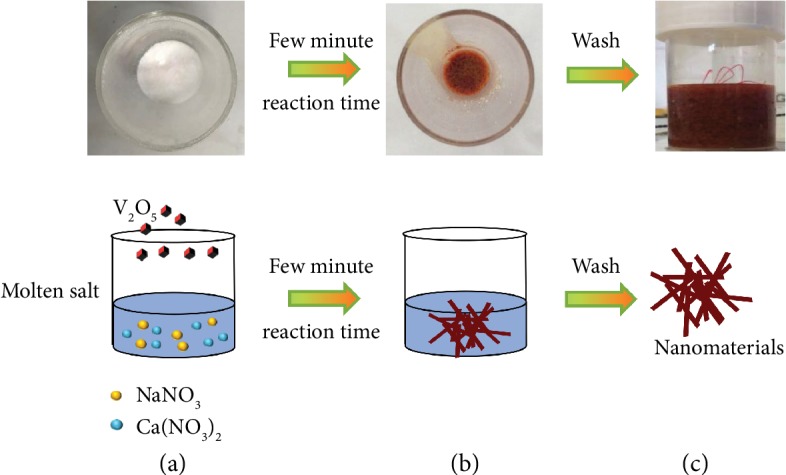
Realistic and schematic representation of the molten salt method synthesis process. (a) The precursor was added when salts were heated to the molten state. (b) The sample was removed from the furnace after reacting for a few minutes. (c) The samples were obtained after washing with DI water.

Low-temperature molten salts are achieved using inorganic salts such as nitrates, sulfates, or chloride. Since salt is in the liquid state, ion diffusion is faster during reaction compared to the conventional solid-state chemistry route—for which high temperature is thus required—resulting in an improvement of the reaction kinetics and homogeneous mixing of reactants. In our experiments, the nitrate was first heated in the air in a 450°C muffle furnace (the furnace was firstly heated to 450°C and then kept at this temperature before adding the nitrates inside) to reach molten state before adding the precursors into it (Figure 1(a)). The precursor was then added inside, and the reaction time was kept to only a few minutes, leading to the formation of a brownish solid well dispersed in the colorless molten salt (Figure 1(b)). After that, the crucible with the product was taken out of the furnace. Once cooled down to room temperature, the recrystallized salt was solubilized in water and the solid product recovered by filtration (Figure 1(c)). More synthesis details have been given in Material Synthesis.

**Figure 2:**
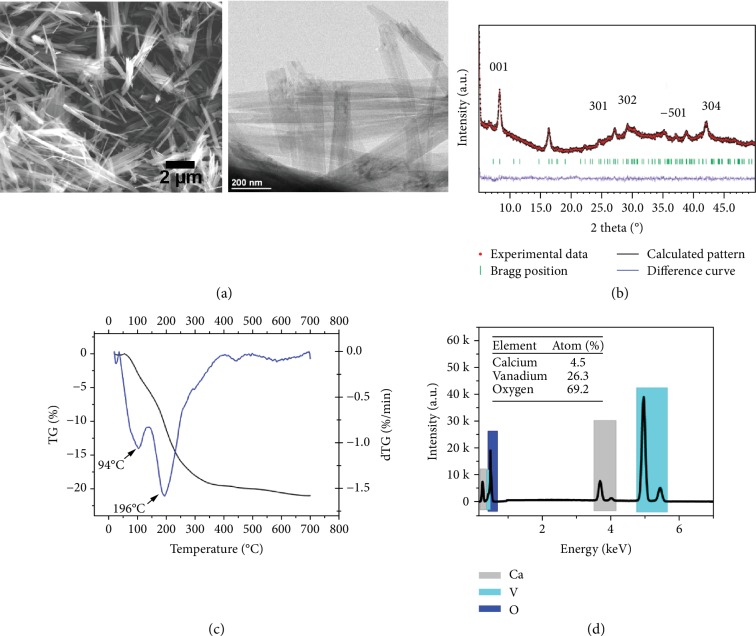
Characterization of CaV_6_O_16_·7H_2_O (CVO) nanomaterial. (a) SEM image and TEM image, (b) X-ray diffraction pattern, (c) TGA result, and (d) EDX image and atom ration of calcium and vanadium element.

1D CVO (Figure 2(a)) compounds were prepared in a mixture of calcium nitrate and sodium nitrate (to decrease the melting point) used as molten salt. SEM image in Figure 2(a) shows the 1D nanoribbon morphology of the prepared CVO with lengths from 2 *μ*m to 10 *μ*m. These nanoribbon structures were highly ordered with uniform shapes and flat surfaces. TEM (Figure 2(a)) studies confirm that the CVO sample adopts a ribbon-like morphology with diameters of 50− 200 nm.

Figure 2(b) shows the X-ray diffraction pattern, where all peaks are well indexed with the CaV_6_O_16_·7H_2_O (JCPDS card number: 01-084-2134); additionally, the weak intensity and broad peak feature evidence a typical nanocrystalline structure of the prepared CVO. The refinement of lattice parameters (*a* = 11.96 Å, *b* = 3.56 Å, and *c* = 10.47 Å) leads to an interlayer spacing of 10.4 Å, which is larger than that of LiV_3_O_8_ (6.36 Å), V_2_O_5_ (4.37 Å) [23], and some other vanadium bronzes incorporating alkali metals.

The hydrated calcium vanadate mineral CaV_6_O_16_·*n*H_2_O has three distinct phases containing 9-, 7-, and 3H_2_O, respectively [25]. The water content in our CVO material was determined using TGA technique in argon atmosphere such as shown in Figure 2(c). The plot reveals a total weight loss of 20.5% from room temperature to 400°C. Two peaks can be clearly observed in the derivative thermos gravimetric (DTG) curve shown in Figure 2(c). As a result, the TG curve can be divided into two parts: from room temperature to 100°C, a smaller weight variation 3.7% weight loss corresponds to physisorbed water, whereas above 100°C, the weight loss of 16.8 wt% is due to the crystal water. This value matches well with the theoretical content in the formula CaV_6_O_16_·7H_2_O (17 wt%). Therefore, the final product can be classified as CaV_6_O_16_·7H_2_O. Several reasons could be explained as the source of the crystal water; the crystal water might be inserted inside the CVO layer during the reaction process or cooling process from moisture or even the washing process [26]. This high content of crystal water might explain the large interlayer spacing of the CVO sample since the interlayer distance of CaV_6_O_16_·7H_2_O (10.4 Å) is larger than that of the partial dehydrated CaV_6_O_16_·3H_2_O (8.08 Å) as reported in [24].

EDX analysis was also achieved to confirm the composition of the CVO particles. As shown in Figure 2(d), peaks located at 0.34 keV, 3.69 keV, and 4.01 keV evidence the presence of Ca, the peaks centered at 0.51 keV, 4.95 keV, and 5.43 keV show the presence of V element while the peak located at 0.53 keV is proof of the existence of the O element. Ca atomic percentage in the sample was estimated at 4.5 ± 0.6%, V content was 26.3 ± 1.1% while O was 69.2 ± 11.6%, leading to a ratio of 1 : 6 : 16 between Ca, V, and O atoms, respectively, which well agrees with the CVO formula. The composition was also verified using X-ray fluorescence spectrometry. As shown in [Supplementary-material supplementary-material-1], a similar Ca : V ratio of 1 : 6 was obtained.

### 2.2. Electrochemical Characterizations

The prepared CVO samples were electrochemically tested in three-electrode Swagelok cells, where an overcapacitive activated carbon electrode was used as a counter electrode and a saturated calomel electrode (SCE) as a reference electrode. The electrolyte was 4.5 M aqueous Ca(NO_3_)_2_ since Lee et al. reported an improved cycling performance of CuHCF in a super-concentrated aqueous electrolyte [27]. However, the low pH value of the 4.5 M aqueous Ca(NO_3_)_2_, measured at 2.3 could result in the partial dissolution of CVO as well as proton (de)intercalation. To tackle these issues, pH was adjusted to 10 by adding Ca(OH)_2_ while additional Ca(NO_3_)_2_·4H_2_O was used to maintain the Ca(NO_3_)_2_ at 4.5 M. A working potential window of ~1.3 V (from -0.7 V to 0.6 V vs. SCE (2.44 to 3.74 V vs. Ca/Ca^2+^) which falls within the H_2_O stability potential window) was obtained during cyclic voltammetry (CV) experiments, achieved at 0.2 mV s^−1^ ([Supplementary-material supplementary-material-1]).

**Figure 3:**
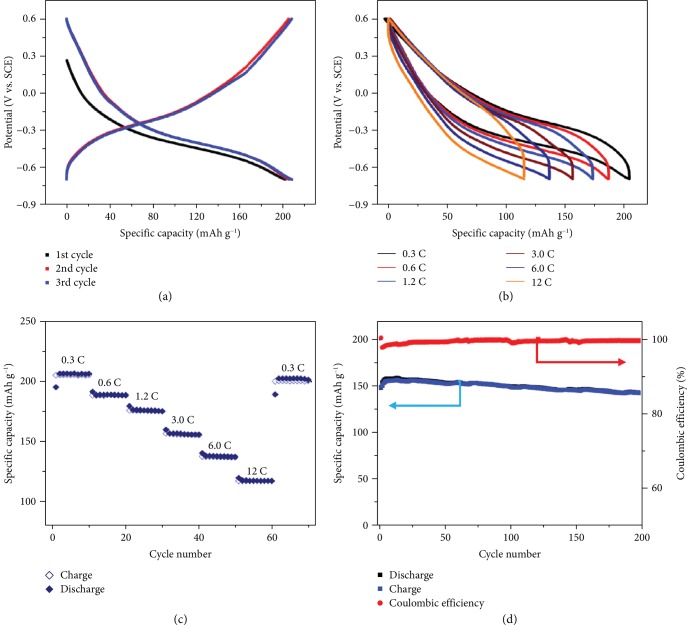
Electrochemical performance of CaV_6_O_16_·7H_2_O (CVO). (a) Galvanostatic charge/discharge profiles at a current density of 0.3 C. (b) Galvanostatic charge/discharge profiles at different current densities. (c) Rate capability at varying C rates. (d) Cycling performance at a current density of 3 C.

The electrochemical characterizations of CVO as the electrode for Ca-ion batteries in the aqueous electrolytes are presented in Figure 3. As shown in Figure 3(a), the initial three charge/discharge galvanostatic plots were almost totally overlapped, suggesting a high reversibility of the Ca^2+^ insertion/extraction process. The initial charge and discharge specific capacities are 203 mA h g^−1^ and 208 mA h g^−1^, respectively, at a current density of 0.25C (1C rate corresponds to a full discharge in one hour that is a current density of 50 mA g^−1^, see [Supplementary-material supplementary-material-1]), corresponding to a 5.5 electron redox process per CaV_6_O_16_·7H_2_O unit. A potential plateau is observed at -0.48 V vs. Ref. during discharge, shifted down to -0.25 V vs. Ref. during charge, resulting in a voltage hysteresis of about -0.23 V, consistent with the CV curves shown in [Supplementary-material supplementary-material-1]. Figure 3(b) shows the charge/discharge galvanostatic plots at various current densities. The voltage hysteresis was only slightly increased when the C rate was 40 times increased, indicating a relatively low activation and concentration polarizations [28]. The electrode rate performance is summarized in Figure 3(c), where the current density was increased stepwise from 0.3C to 12C and then returned to 0.3C. Upon continuous cycling under various current densities, the specific capacities dropped only marginally on doubling/quadrupling the rates and immediately recovered with their reversal. The initial discharge capacity of 205 mA h g^−1^ at 0.3C had only a small decrease of 15% (178 mA h g^−1^) at 1.2C, and 117 mA h g^−1^ was still delivered at 12C rate, thus demonstrating the good power capability of the CVO electrode. This can be explained by the nanoribbon morphology, which increases the active material/electrolyte interface area as well as shortens diffusion distances. The cycle life of a CVO electrode was evaluated at the 3C rate for 200 cycles (Figure 3(d)). The electrode delivers a reversible capacity of 141 mA h g^−1^ with 97% capacity retention after 200 cycles. During the initial 12 cycles, the battery exhibits gradually increasing capacity until reaching a maximum capacity of 157 mA h g^−1^ and then declines slowly for the next 188 cycles. Moreover, high initial coulombic efficiency (98%) was observed, and nearly 100% coulombic efficiency can be achieved after just 8 cycles, which further proves the superior performance of CVO in aqueous Ca-ion batteries. The long cycling stability might be explained in that the volume expansion/contraction during charge/discharge cycles and the associated material fading can be effectively avoided with 1D-nanostructured architecture and that this raises the cycling stability [29]. Besides, the crystal water between the CVO layers might also play a significant role in affecting the electrochemical performances. First of all, the interlayer spacing can be further expanded by the intercalation of water molecules, providing an increased Ca^2+^ diffusion rate [30]. Besides, according to Zhang et al. [23], the interlayer water molecules form O-O bonds with the V_6_O_16_ layers. In other words, interlayer water molecules could be taken as pillars to alleviate the structural strain thus maintaining substantial architectural stability providing a higher cyclability. Additionally, interlayer water molecules could effectively screen the electrostatic interactions between Ca^2+^ and the host framework or the CVO layers to facilitate Ca^2+^ diffusion [30].

To the best of our knowledge, the high reversible specific capacity, rate capacity, and cycling stability of these CVO nanoribbons compare positively with previously reported materials for aqueous Ca-ion batteries (see [Supplementary-material supplementary-material-1]) such as K_0.31_MnO_2_·0.25H_2_O [12] and K_0.3_CuHCF [13].

Interestingly, the molten salt synthesis route was extended to the family of 1D *M*V_3_O_8_ (*M* = Li, K) ([Supplementary-material supplementary-material-1]) which also shows nanoribbon morphology and own excellent electrochemical performance. For example, nonhydrated LiV_3_O_8_ (JCPDS card number: 35-0437) synthesized by MSM was used as a cathode material for the Li-ion battery. High capacity of 310 mA h g^−1^ at 100 mA g^−1^ and 170 mA h g^−1^ at 2 A g^−1^ could be achieved without any optimization in a nonaqueous electrolyte ([Supplementary-material supplementary-material-1]). These performances compare positively with those of LiV_3_O_8_ nanoribbons synthesized by electrospinning method [28]. 1D CaV_2_O_6_ ([Supplementary-material supplementary-material-1]) was also first proposed as an electrode material for the aqueous Ca-ion battery, with a capacity of 153 mA h g^−1^, long cycle life (>95% capacity retention after 700 cycles at a 5C rate), and high-rate performance (58 mA h g^−1^ at 50C) during Ca-ion (de)intercalation reactions ([Supplementary-material supplementary-material-1]). By playing with the nature of the precursor, the reaction time, and the heating temperature, K_3_V_5_O_14_ and Ca_2_V_2_O_7_ were also successfully prepared by MSM with high yield as well as CaMoO_4_ ([Supplementary-material supplementary-material-1]). As can be seen, all the synthesized compounds (CaV_6_O_16_·7H_2_O, CaV_2_O_6_, LiV_3_O_8_, and KV_3_O_8_) own similar layered structures where ions intercalated between the layers. The main difference lies in the bonds between the different species (ionocovalent V-O, ionic Ca-O, and van der Walls interaction for H_2_O) as well as the way the different VO_6_ octahedra and VO_5_ square pyramids are connected to build the V_3_O_8_ layers inducing anisotropy in the growing kinetic. In genreal, layered structure can easily form 2D morphologies. However, in the case of these V-based compounds, 1D nanoribbon morphology forms are due to preferential, fast growth of the V_3_O_8_ or V_2_O_6_ layer in one specific direction. For example, 1D nanoribbon morphology is obtained for CaV_6_O_16_·7H_2_O because of the rapid growth in the [100] direction of the alternate strings of V_4_O_11_ and V_2_O_6_, with a poor connection between each other. The short time of the molten salt synthesis process with exacerbated kinetic differences is assumed to be at the origin of the nanoribbon-like morphology observed from different samples by favoring one-direction growth mechanism.

To sum up, the present molten salt route offers clear advantages over previous preparation procedures in terms of time and offers new opportunities to design electrode materials for energy storage applications. Furthermore, the high synthesis yield of the molten salt-based method is compatible with large-scale preparation.

### 2.3. Charge Storage Mechanism

Differently from nonaqueous systems, the charge storage mechanism in the aqueous electrolyte is more complex because of the possible contribution of proton or hydronium ions. For instance, Sun et al. report the simultaneous H^+^ and Zn^2+^ insertion/deinsertion reactions at the positive electrode of an aqueous Zn-MnO_2_ battery [31]. Hyoung et al. studied K_0.31_MnO_2_·0.25H_2_O as a cathode material in aqueous Ca-ion batteries [12]. They confirmed that Ca^2+^ was the main contributor to the electrochemical reaction while hydronium ions were also proposed to cointercalate during the reaction [12].

**Figure 4:**
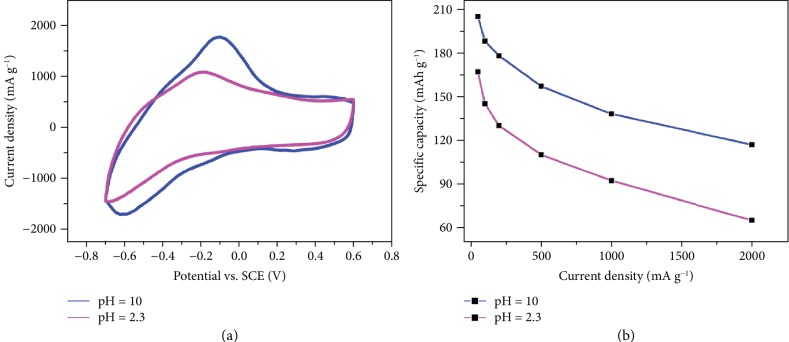
Electrochemical behavior of a CaV_6_O_16_·7H_2_O (CVO) electrode in pH = 2.3 (4.5 M Ca(NO_3_)_2_) and pH = 10 (4.5 M Ca(NO_3_)_2_+Ca(OH)_2_) electrolytes, with (a) CV plots recorded at a potential scan rate of 2 mV s^−1^ and (b) comparison of the discharged (reduction process) specific capacity versus various current density of the CVO electrode in the two different electrolytes.

In this work, to clear out the role of hydronium ions, CaV_6_O_16_·7H_2_O electrodes were tested in various electrolytes with the same Ca(NO_3_)_2_ concentration but at different pH, by increasing the pH with Ca(OH)_2_. The similar electrochemical signatures of the CV (Figure 4(a)) in pH 2.3 and pH 10 electrolytes support a redox reaction where the main process is the intercalation of the Ca ion in the CVO host structure, according to

(1) $$\begin{array}{c} {\text{CaV}}_{6}{\text{O}}_{16}\cdot 7{\text{H}}_{2}\text{O}+2.75\text{ C}{\text{a}}^{2+}+5.5\;{\text{e}}^{-}↔{\text{Ca}}_{3.75}{\text{V}}_{6}{\text{O}}_{16}\cdot 7{\text{H}}_{2}\text{O} \end{array}$$

Besides, the capacity of CVO in the pH 10 electrolyte was found to be slightly higher, where the proton or hydronium ion concentration is negligible.  Figure 4(b) compares the discharge capacity in both electrolytes at various current densities, showing better capacity retention in the pH 10 electrolyte than in pH 2.3 (59% and 38%, respectively) from 50 mA g^−1^ to 2000 mA g^−1^. All these features support the Ca-ion intercalation as the main charge storage mechanism during the charge/discharge processes of the CVO electrode in Ca^2+^-containing aqueous electrolyte at pH 10.

An EQCM study was achieved using the CVO electrode in the two electrolytes (pH = 2.3 and pH = 10), to get further information on the charge storage mechanism (see [Supplementary-material supplementary-material-1]). Results confirmed that a heavier ion was intercalated in the pH = 10 electrolyte compared with the acidic one (pH = 2.3). As a result, the heavier molar weight obtained for the pH = 10 electrolyte would support the intercalation of a heavier Ca^2+^ ion with one water molecule, while the intercalation of hydronium ions would explain the charge storage mechanism in the acidic electrolyte. Those results also exclude the possibility for CVO dissolution since the frequencies at the starting and ending points of the CV are roughly the same (see [Supplementary-material supplementary-material-1]).

### 2.4. Reaction Kinetics

**Figure 5:**
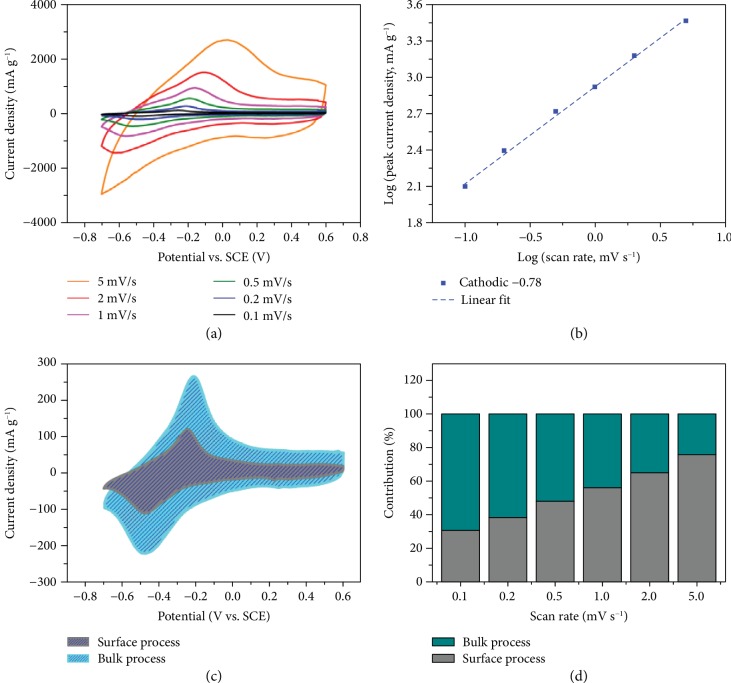
Reaction kinetic analysis of CaV_6_O_16_·7H_2_O (CVO). (a) Cyclic voltammetry curves at various scan rates from 0.05 to 5 mV s^−1^ in 4.5 M Ca(NO_3_)_2_ at pH 10 electrolyte. (b) The b-coefficient calculated from the peak current and scan rate. (c) Separation of the surface (nondiffusion-limited) and bulk (diffusion-limited) processes of charge storage at 0.2 mV s^−1^. (d) Surface and bulk current contributions to the total charge versus the scan rates from 0.05 to 5 mVs^−1^.

To further understand the electrochemical kinetics of CVO in Ca-ion batteries, CVO electrodes were tested at various potential scan rates from 0.1 to 5 mV s^−1^ in a pH 10 electrolyte. There is mainly one set of redox peaks observed at -0.25/-0.48 V at a 0.2 mV s^−1^ scan rate (Figure 5(a)), representing the dominant electrochemical processes assumed to involve the V^5+^/V^4+^ redox couple. Even though the characteristic peaks turn broader and the peak separation increases with increasing potential scan rate, the CV curves show similar signatures even at 5 mV s^−1^ corresponding to discharge in 260 s, equivalent to 14C (Figure 5(a)). The kinetic analysis has been made following the method proposed by Wang et al. [32], where the peak current is plotted versus the potential scan rate to calculate the *b* value:

(2) $$\begin{array}{c} i=\text{a}v^{\text{b}}, \end{array}$$

where *i* is the peak current of various scan rates and *v* is the scan rate. The *b* value is the slope of the fitting line with peak current versus scan rate in the log scale. The *b* value is between 0.5 and 1; a *b* value of 1 represents a charge storage dominated by surface processes whereas the diffusion-controlled process gives a minimum *b* value (*b* = 0.5) [33]. As seen in Figure 5(b), the average *b* value is 0.70 for the cathodic peak and 0.71 for the anodic peak, which likely indicates that both contributions (surface and bulk) are present. The surface (changing with *v*) and bulk (changing with *v*^1/2^) contributions to the total current were calculated using the following equation, proposed by Wang's group [32]:

(3) $$\begin{array}{c} i\left(v\right)=k_{1}v+k_{2}v^{1/2}. \end{array}$$

Figure 5(c) shows the contribution of surface vs. bulk processes obtained from equation (3) during the CV achieved at 0.2 mV s^−1^. While the electrochemical storage in CVO is mainly controlled by the solid-state diffusion process at a low scan rate (62%), the surface process accounts for about 38% of the total current. Figure 5(d) shows the change of the surface and bulk contributions at various potential scan rates. As expected, the capacitive charge contribution increases with increasing potential sweep rate, reaching a maximum value of 77% at 5 mV s^−1^. The above data shows that the surface intercalation redox process (not diffusion-limited) plays a significant role in the electrochemical reaction, which accounts for the excellent rate capability of the present CVO electrode since the bulk intercalation process is diffusion-limited at high current density.

**Figure 6:**
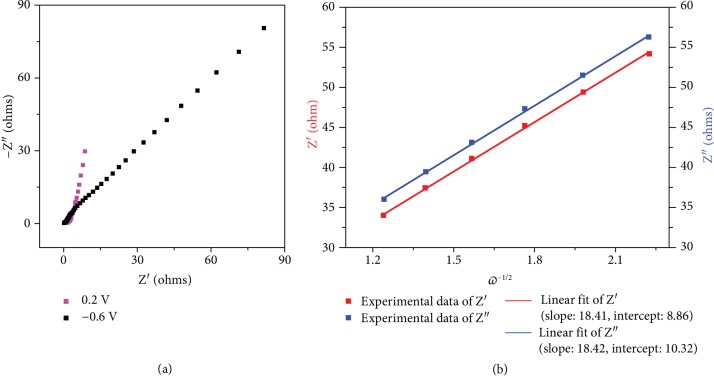
Electrochemical impedance spectroscopy measurements of a CaV_6_O_16_·7H_2_O (CVO) electrode in 4.5 M Ca(NO_3_)_2_ at pH 10 electrolyte. (a) EIS analysis at different potentials at the 3^rd^ discharge (reduction) cycle. (b) Change of the real part of the impedance *Z*′ versus the square root of the reverse of the pulsation *ω*^−1/2^ in the low-frequency region (from 10 to 32 mHz) recorded at -0.6 V during the 3^rd^ discharge cycle.

Electrochemical impedance spectroscopy (EIS) measurements were also done to get a further understanding of the reaction process. The CVO electrode was discharged stepwise from OCV to -0.6 V vs. Ref. and then charged back to 0.2 V vs. Ref.. This process was repeated twice following the same procedure before achieving EIS measurements. The Nyquist plots recorded at various constant potentials during discharge (reduction) are shown in Figure 6(a); [Supplementary-material supplementary-material-1] shows the Nyquist plots recorded during the charge (oxidation) step. In the high-frequency region, the Nyquist plots normally show two different behaviors depending on the applied potential. At 0.2 V vs. Ref., the impedance plot shows a typical blocking electrode behavior; this is consistent with the “double layer-like” signature observed in the CV of Figure 5 above. At -0.6 V vs. Ref., the increase of the impedance in the low-frequency region evidences a diffusion-limited process [34], in agreement with the redox reaction visible on the CV in Figure 5.

Ca-ion diffusion coefficient into the structure was calculated from the impedance measured at -0.6 V.  Figure 6(b) shows the change of the real part of the impedance with *ω*^−1/2^. The linear change of the impedance vs. *ω*^−1/2^ in the low-frequency region results from the diffusion-controlled electrochemical reaction kinetics [35]. The diffusion coefficient can be calculated according to the following equation [35]:

(4) $$\begin{array}{c} D=\frac{1}{2}{\left(\frac{RT}{A{C\sigma z}^{2}F^{2}}\right)}^{2}, \end{array}$$

where *A* is the surface area of the electrode, *z* is the valence of the ion, *F* is the Faraday constant, *C* is the concentration of Ca^2+^ in the CVO electrode, *R* is the gas constant, *T* is the room temperature in our experiment, and *σ* is the slope of the line *Z*′∼*ω*^−1/2^ in the low-frequency region, which is obtained from Figure 6(b).

Ca-ion diffusion coefficient is calculated to be 1.8∗10^−10^ cm^2^ s^−1^ at -0.6 V, which is similar to that obtained for Ca_0.25_V_2_O_5_ used as the cathode in a Zn-ion battery [24]. This large value can be explained by the 1D morphology and the large interlayer distance of our CVO material. It is two decades higher than the Li^+^ diffusion in LiV_3_O_8_ [28], TiO_2_ [36], and LiCoO_2_ [37] in organic or aqueous cells, which shows that the diffusion of Ca ion in the solid CVO electrode might not be the rate-limiting step of the mass transport.

Finally, the set of results presented here highlights the high performance of CaV_6_O_16_·7H_2_O (CVO) with 1D morphology as an electrode material for Ca-ion aqueous battery application, prepared from the molten salt method synthesis route. Beyond CVO, this method allows for the preparation of other 1D materials with high electrochemical performance, which highlights the opportunities offered by such a method to prepare materials with improved performance for energy storage applications.

## 3. Conclusions

In this paper, we proposed a general and rapid molten salt synthesis route (MSM) to prepare various 1D cation-intercalated vanadium-based ternary metal oxides. Nanoribbons LiV_3_O_8_, CaV_2_O_6_ and CVO have also been prepared and used as electrode materials and excellent electrochemical performances were observed. As a novel electrode material for aqueous Ca-ion batteries, the electrochemical performance and storage mechanism of CVO were studied in detail. Favorable structural features and nanoscale morphology act in concert to enable high specific capacities (205 mA h g^−1^), high-rate kinetics (117 mA h g^−1^ at 12C), and long-term cyclability (97% capacity retention after 200 cycles at 3.0C). Additionally, 1D CaV_2_O_6_ was also first proposed as the electrode material for the aqueous Ca-ion battery, with a capacity of 153 mA h g^−1^, long cycle life (>95% capacity retention after 700 cycles at a 5C rate) and high-rate performance (58 mA h g^−1^ at 50C) during Ca-ion (de)intercalation reactions.

## 4. Materials and Methods

### 4.1. Material Synthesis

Ca(NO_3_)_2_·4H_2_O (99%), LiNO_3_ (99%), NaNO_3_ (99%), and KNO_3_(99%) were obtained from Sigma-Aldrich and V_2_O_5_ (99.6%) from Aldrich and VCl_3_(97%) from Alfa Aesar.

In a typical procedure, 6 g of nitrate powder (Ca(NO_3_)_2_·4H_2_O : NaNO_3_ = 1 : 1 in weight ratio) mixed in a quartz crucible is transferred into the furnace at a temperature of 450°C in the air to get colorless molten salt. 0.2 g of V_2_O_5_ powder is then added in the molten salt. After 3.5 minutes of holding time, the product inside the crucible is quenched in the air. The as-synthesized product was thoroughly washed with distilled water to remove excess nitrate powder and finally freeze-dried. *M*V_3_O_8_ (*M* = Li, K) was prepared following the same molten salt process but using 5 g of LiNO_3_ or 5 g of KNO_3_. The molten salt is obtained by heating at 380°C. 0.2 g of VCl_3_ used as the vanadium source is introduced in the molten salt and the reaction time is set to 1 minute. To prepare K_3_V_5_O_14_, 5 g of KNO_3_ was heated at 380°C while 0.2 g of V_2_O_5_ was used as the vanadium source; the reaction time is only 1 minute. For CaV_2_O_6_ and Ca_2_V_2_O_7_, the composition changes with the increase of reaction time; a mixture containing 3 g of KNO_3_ and 3 g Ca(NO_3_)_2_·4H_2_O was heated as a solvent at 480 °C while 0.2 g of V_2_O_5_ was used as the vanadium source—the reaction times are 5 minutes and 10 minutes, respectively. The washing and drying procedure is the same as previous.

### 4.2. Physical Characterization

The X-ray diffraction (XRD) pattern is collected using a D4 ENDEAVOR diffractometer (Bruker, Germany) equipped with Cu K*α* radiation (*λ* = 0.154 nm). Data are collected in the 5 to 50 2*θ* angle range using 0.0099° steps in 1 hour. The patterns are refined using the profile matching method implemented in the Jana2006 software.

The composition of the samples was examined by an X-ray fluorescence spectrometer BRUKER S2 Ranger. The morphology and composition of the sample were observed with a Scanning Electron Microscope (SEM) JSM 7100F (JEOL, Japan) at an accelerating voltage of 20 kV with energy-dispersive X-ray spectroscopy (EDX) capabilities. Transmission Electron Microscopy (TEM) and High-Resolution Transmission Electron Microscopy (HTEM) images were performed using a JEM-2100F microscope working at an acceleration voltage of 200 kV. Thermo gravimetric analysis (TGA) was performed in an alumina crucible using an ATG-ATD Setaram TGDTA 92 in nitrogen using a ramping rate of 10°C min^−1^.

### 4.3. Electrochemical Measurements

For the electrochemical performance evaluation, 3-electrode Swagelok© cells were assembled in ambient conditions. In this setup, the counter electrode was YP-50 (Kuraray, Japan) and the reference electrode was the saturated calomel electrode (SCE). The R-XR110 type SCE reference electrode was purchased from Bio-Logic Science Instruments. To prepare the electrolyte, 4.5 M Ca(NO_3_)_2_ was dissolved in deionized water firstly and then saturated Ca(OH)_2_ was added inside to increase the pH until 10. Finally, additional Ca(NO_3_)_2_·4H_2_O was added to ensure that the concentration of Ca(NO_3_)_2_ was still kept at 4.5 M. The electrolytes were purged with argon gas before use. The separator was two layers of 260 *μ*m thick porous borosilicate glass fibers (Whatman GF/A). To prepare the working electrode, 70 wt% active material and 20 wt% conductive carbon (Super-P) were mixed homogeneously first and then added inside a bottle containing 10 wt% of polyvinylidene fluoride binder in N-methyl-2-pyrrolidone. The active material mass loading is around 2–4 mg cm^−2^. A multichannel VMP3 electrochemical working station (Bio-Logic, S.A.) has been applied for all the electrochemical tests. The electrochemical impedance spectroscopy measurement was scanned from 1 MHz to 0.01 Hz.

For sample preparation of the electrochemical quartz crystal microbalance (EQCM), Bio-Logic 1 in. diameter Au-coated quartz crystals (oscillating frequency, f0, 5 MHz) were coated using a precise pipette (Gilson PIPETMAN Classic P20) with a slurry containing 70 wt% of active material CVO powder, 20 wt% of conducting carbon (Super-P), and 10 wt% of polyvinylidene fluoride (Arkema, CAS #24937-79-9) binder in N-methyl-2-pyrrolidone (Sigma-Aldrich, CAS #872-50-4). The coated quartz crystal was placed on a PTFE holder in which the coated side is orientated toward the reference and the counter electrode served as the working electrode in a 3-electrode electrochemical cell. The counter electrode is a platinum-coated titanium mesh. The SCE was used as a reference electrode placed between working and counter electrodes. Three electrodes were set in glassware and immersed in 4.5 M Ca(NO_3_)_2_ (pH = 2.3) or a mixture of Ca(NO_3_)_2_ and Ca(OH)_2_ (pH = 10) aqueous electrolytes. All the EQCM electrochemical measurements were carried out by a Maxtek EQCM system combined with an Autolab PGSTAT101 which was used for simultaneous EQCM and electrochemical measurements.

The EQCM data was treated based on the Sauerbrey equation: Δ*m* = −Cf∗Δ*f*, where Δ*m* is the change of mass of the coating and Cf is the sensitivity factor of the crystal. The sensitivity factor of the coated quartz was obtained by performing a copper deposition experiment conducted in 0.1 M CuSO_4_ mixed with 1 M H_2_SO_4_ by applying a constant current of 5 mA for 120 seconds. In this experiment, the Cf was calculated to be 5.08 ng·Hz^−1^ (or 4.05 ng·Hz^−1^ cm^−2^ taking into account the Au crystal electrode surface of 1.27 cm^2^). For consistent results, few cycles were run before starting EQCM measurements, to start from stable, reproducible electrochemical signatures.

## Supplementary Materials

Supplementary Materials Table S1: compare the different properties of monovalent ions and multivalent ions. Figure S1: the result of the X-ray fluorescence spectrometer measurement of CaV_6_O_16_·7H_2_O (CVO) sample. (a) Wavelength-dispersive XRF spectrum with shoulder peak of Ca element and V element; (b) the atom ratio between Ca element and V element. Figure S2: initial five first CV cycles of CaV_6_O_16_·7H_2_O (CVO) recorded at 0.2 mV s^−1^ in 4.5 M Ca(NO_3_)_2_ aqueous electrolyte with Ca(OH)_2_ to adjust the pH at 10. Figure S3: charge/discharge curves of CaV_6_O_16_·7H_2_O (CVO) tested at different current densities in 4.5 M Ca(NO_3_)_2_ electrolyte containing Ca(OH)_2_ to adjust the pH at 10. (a) Galvanostatic charge/discharge curve at 50, 100, and 200 mA g^−1^; (b) galvanostatic charge/discharge curve at 500, 1000, and 2000 mA g^−1^. Table S2: summary of electrochemical performance reported for various Ca-ion intercalation materials compared to CaV_6_O_16_·7H_2_O (CVO) showing higher specific capacity and better cycling stability. Figure S4: X-ray diffraction pattern of (a) KV_3_O_8_ and (b) LiV_3_O_8_; SEM graph of (c) KV_3_O_8_ and (d) LiV_3_O_8_. The corresponding XRD pattern in Figure S5a can be indexed to a well-crystallized layered KV_3_O_8_ (JCPDS 22-1247); the intensity of the (100) peak is extremely high, suggesting that the (100) planes are probably the major growth direction. Figure S5c confirmed that the KV_3_O_8_ had nanofiber features with a length of 10–20 *μ*m and diameters of around 100 nm. As shown in Figure S5d, the monoclinic LiV_3_O_8_ (JCPDS 35-0437, space group: P21/m) had typical diameters of around 200 nm and the nanowire morphology. Figure S6: characterization of CaV_2_O_6_: (a) X-ray diffraction pattern and (b) SEM image. Figure S7: electrochemical performance of CaV_2_O_6_: (a) first five CV cycles recorded at 0.2 mV s^−1^. (b) Galvanostatic charge/discharge profiles at different current densities. (c) Rate capability at varying C rates. (d) Cycling performance at a current density of 5C. Figure S8: X-ray diffraction pattern of (a) K_3_V_5_O_14_, (b) CaV_2_O_6_, (c) Ca_2_V_2_O_7_, and (d) CaMoO_4_. Figure S9: CV and EQCM frequency response (a) in pH = 2.3 aqueous electrolyte at 20 mV s^−1^ and (b) in pH = 10 aqueous electrolyte at 10 mV s^−1^. (c) CV of CVO in pH = 10 aqueous electrolyte and the corresponding mass change at 10 mV s^−1^. (d) Electrode mass change vs. charge during the polarization of CVO in pH = 2.3 (4.5 M Ca(NO_3_)_2_) and pH = 10 (4.5 M Ca(NO_3_)_2_+Ca(OH)_2_) electrolytes at 10 mV s^−1^. Figure S10: EIS analysis at different potentials at the fifth charge (oxidation) cycle.
