# Supplementary material for: Ultrafast Synthesis of Calcium Vanadate for Superior Aqueous Calcium-Ion Battery
Source: Research (Wash D C). 2019 Nov 29;2019:6585686. doi: 10.34133/2019/6585686 (PMC6944483; doi:10.34133/2019/6585686)
Supplement: Supplementary Materials — Table S1: compare the different properties of monovalent ions and multivalent ions. Figure S1: the result of the X-ray fluorescence spectrometer measurement of CaV6O16·7H2O (CVO) sample. (a) Wavelength-dispersive XRF spectrum with shoulder peak of Ca element and V element; (b) the atom ratio between Ca element and V element. Figure S2: initial five first CV cycles of CaV6O16·7H2O (CVO) recorded at 0.2 mV s−1 in 4.5 M Ca(NO3)2 aqueous electrolyte with Ca(OH)2 to adjust the pH at 10. Figure S3: charge/discharge curves of CaV6O16·7H2O (CVO) tested at different current densities in 4.5 M Ca(NO3)2 electrolyte containing Ca(OH)2 to adjust the pH at 10. (a) Galvanostatic charge/discharge curve at 50, 100, and 200 mA g−1; (b) galvanostatic charge/discharge curve at 500, 1000, and 2000 mA g−1. Table S2: summary of electrochemical performance reported for various Ca-ion intercalation materials compared to CaV6O16·7H2O (CVO) showing higher specific capacity and better cycling stability. Figure S4: X-ray diffraction pattern of (a) KV3O8 and (b) LiV3O8; SEM graph of (c) KV3O8 and (d) LiV3O8. The corresponding XRD pattern in Figure S5a can be indexed to a well-crystallized layered KV3O8 (JCPDS 22-1247); the intensity of the (100) peak is extremely high, suggesting that the (100) planes are probably the major growth direction. Figure S5c confirmed that the KV3O8 had nanofiber features with a length of 10–20 μm and diameters of around 100 nm. As shown in Figure S5d, the monoclinic LiV3O8 (JCPDS 35-0437, space group: P21/m) had typical diameters of around 200 nm and the nanowire morphology. Figure S6: characterization of CaV2O6: (a) X-ray diffraction pattern and (b) SEM image. Figure S7: electrochemical performance of CaV2O6: (a) first five CV cycles recorded at 0.2 mV s−1. (b) Galvanostatic charge/discharge profiles at different current densities. (c) Rate capability at varying C rates. (d) Cycling performance at a current density of 5C. Figure S8: X-ray diffraction pattern of (a) [file 6585686.f1.docx]

**SUPPORTING INFORMATION**

**Ultrafast synthesis of Calcium Vanadate for superior aqueous Calcium-Ion Battery**

Liyuan Liu^1,2^, Yih-chyng Wu^1,2^, Patrick Rozier^1,2^, Pierre-Louis Taberna^1,2^ and Patrice Simon^1,2^*

^1^ CIRIMAT, UMR CNRS 5085, Université Paul Sabatier Toulouse III, 118 route de Narbonne, 31062 Toulouse, France

^2^ RS2E, Réseau Français sur le Stockage Electrochimique de l’Energie, FR CNRS 3459, 80039 Amiens Cedex, France.

* corresponding author: simon@chimie.ups-tlse.fr

**Table S1** Comparison of different characteristics of monovalent and multivalent ions.


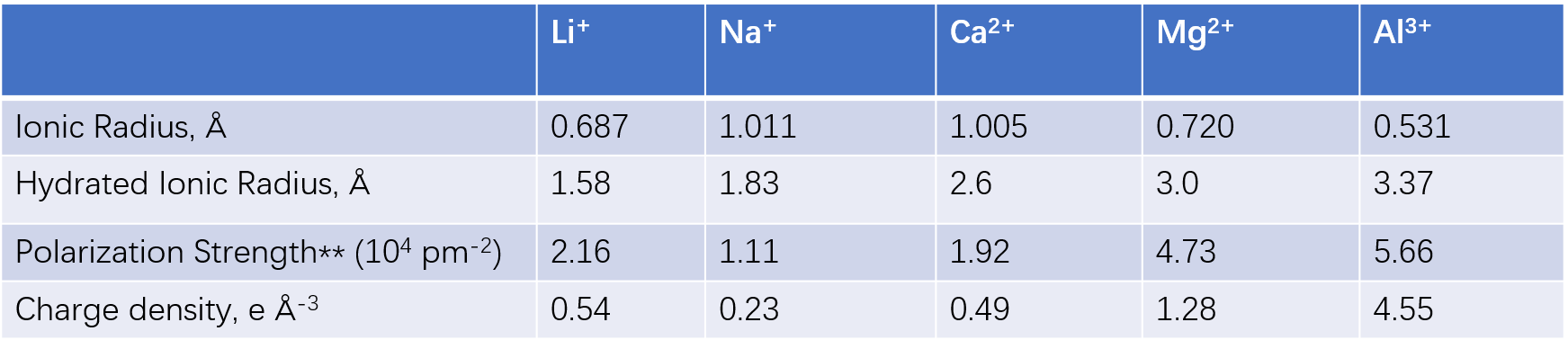


**Polarization strength (*P*) is calculated as *P* = *q r*^-2^, where *q* is the charge number of the cation and *r* is the ion radius.


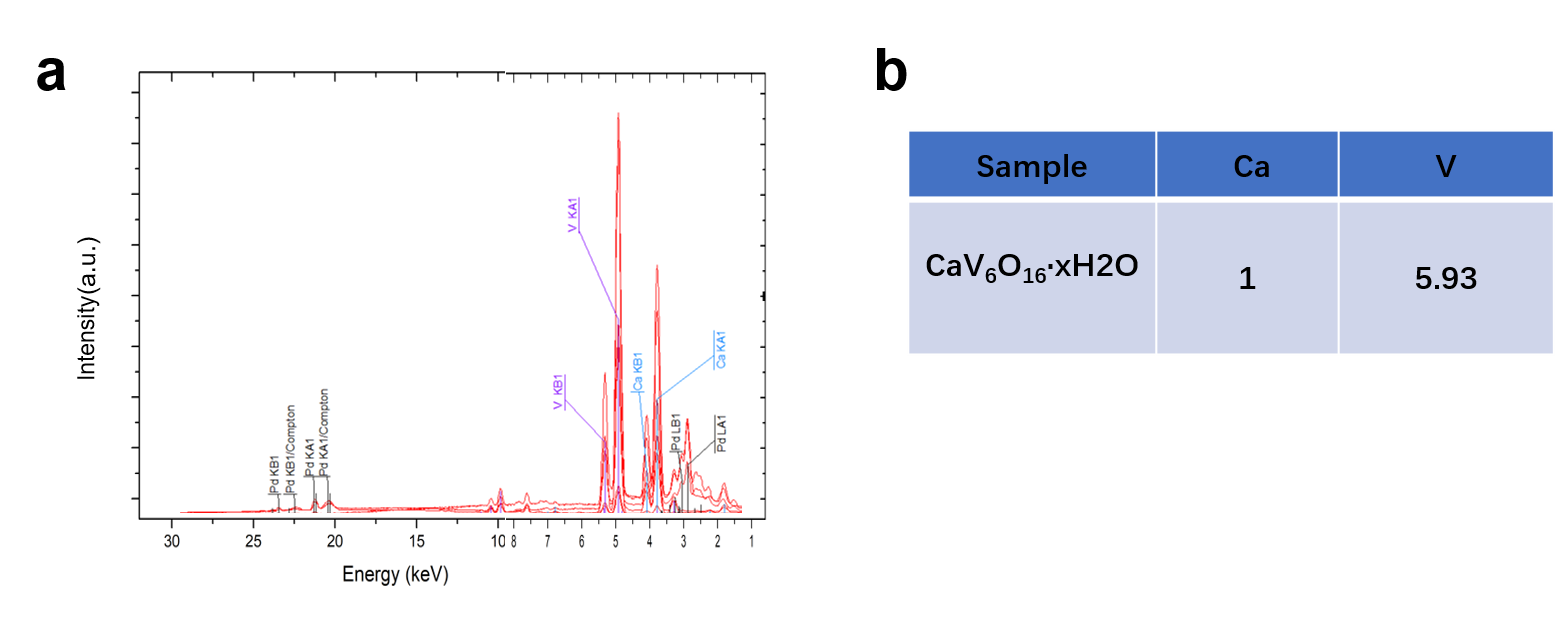


**Figure S1:** The result of X-ray fluorescence spectrometer measurement of CaV_6_O_16_·7H_2_O (CVO) sample. (a) Wavelength dispersive XRF spectrum with shoulder peak of Ca element and V element; (b) The atom ratio between Ca element and V element.


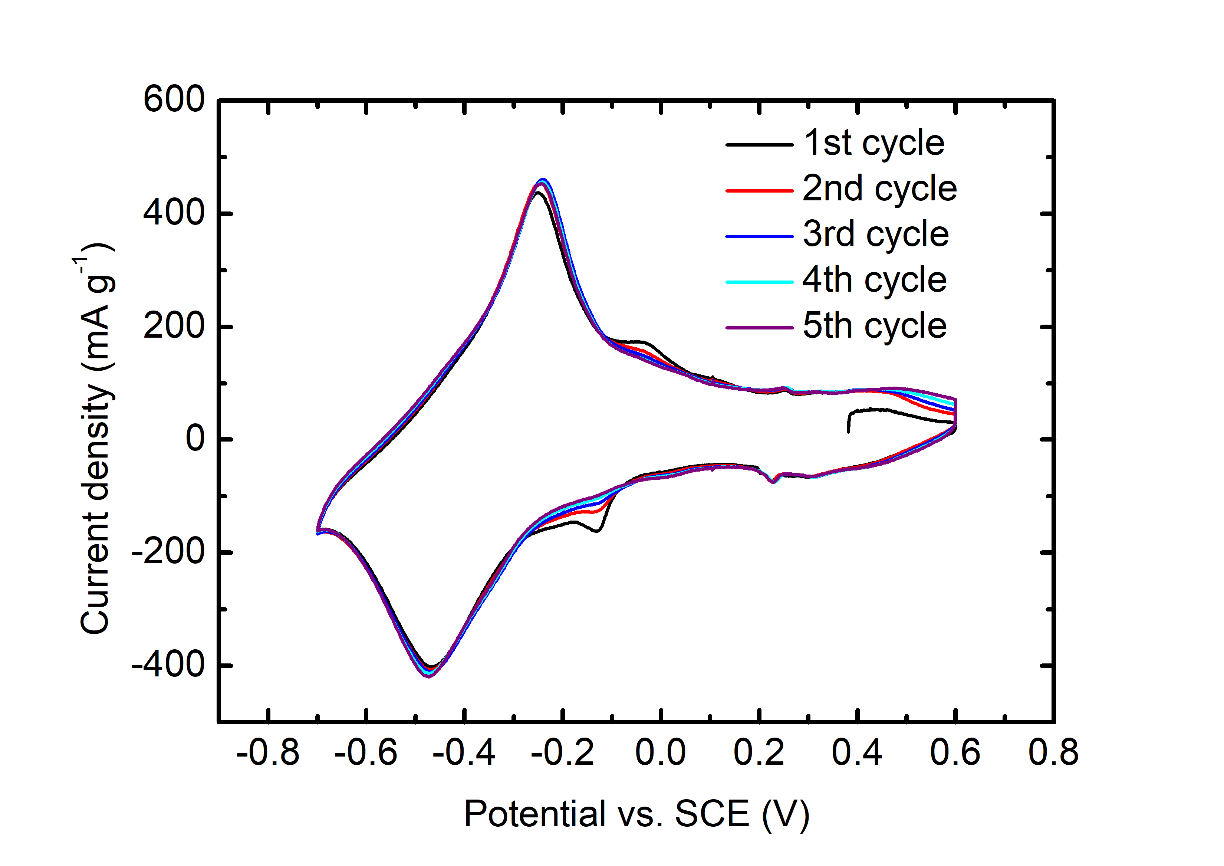


**Figure S2:** Initial five first CV cycles of CaV_6_O_16_·7H_2_O (CVO) recorded at 0.2 mV s^-1^ in 4.5 M Ca(NO_3_)_2_ aqueous electrolyte with Ca(OH)_2_ to adjust the pH at 10.


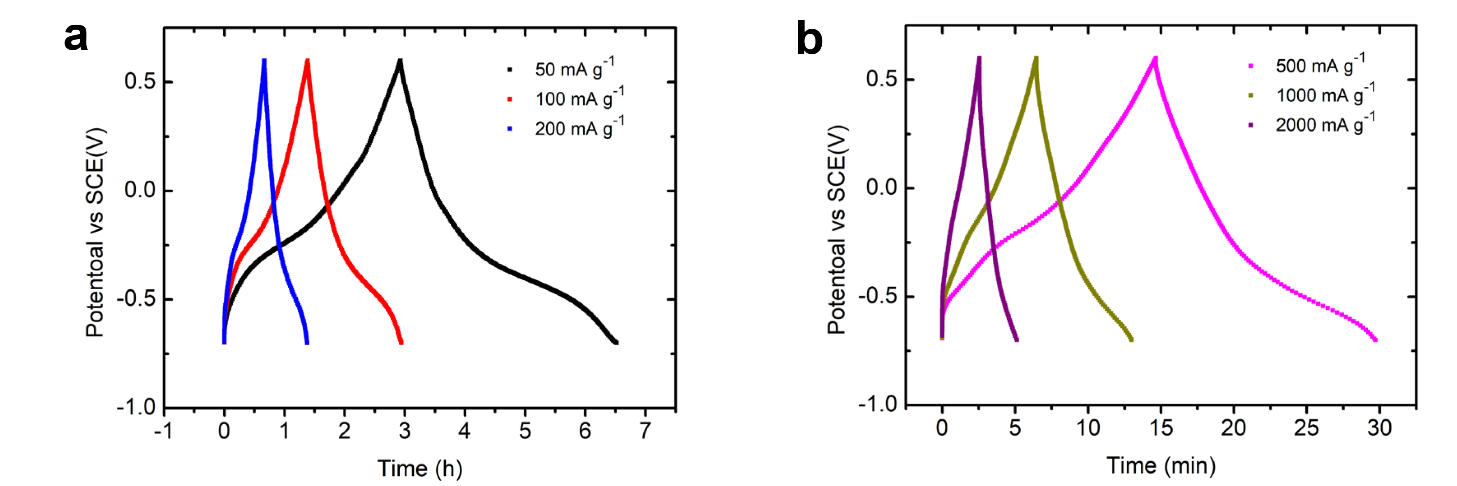


**Figure S3:** Charge-discharge curves of CaV_6_O_16_·7H_2_O (CVO) tested at different current densities in 4.5M Ca(NO_3_)_2_ electrolyte containing Ca(OH)_2_ to adjust the pH at 10. (a) Galvanostatic charge/discharge curve at 50, 100, 200 mA g^-1^; (b) Galvanostatic charge/discharge curve at 500, 1000, 2000 mA g^-1^.

**Table S2**: Summary of electrochemical performance reported for various Ca-ion intercalation materials compared to CaV_6_O_16_·7H_2_O (CVO) showing higher specific capacity and better cycling stability.


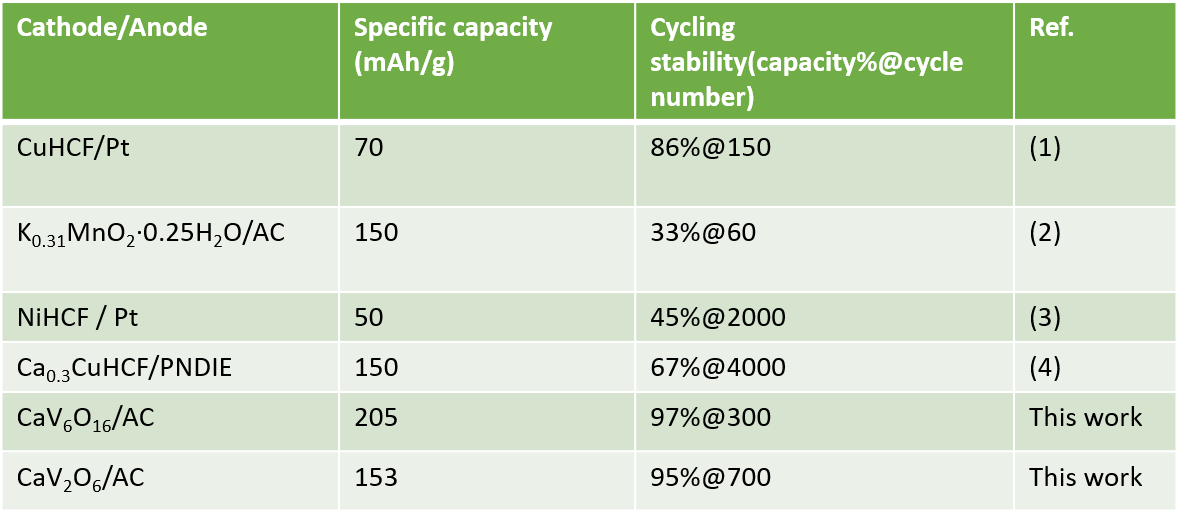


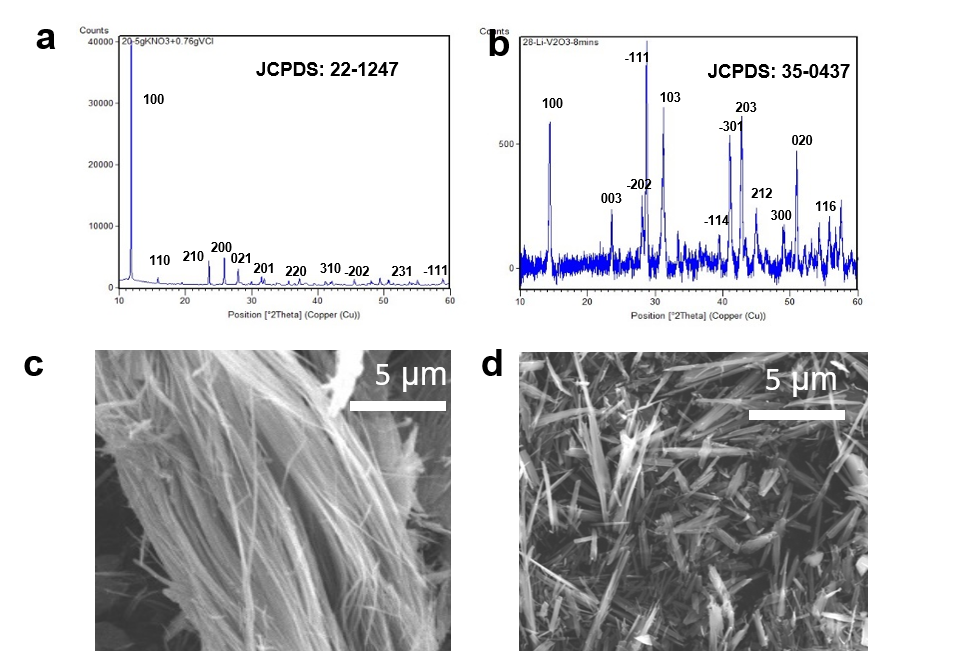


**Figure S4:** X-ray diffraction pattern of (a) KV_3_O_8_ and (b) LiV_3_O_8_; SEM graph of (c) KV_3_O_8_ and (d) LiV_3_O_8_. The corresponding XRD pattern in Figure S5a can be indexed to well-crystallized layered KV_3_O_8_ (JCPDS 22-1247), the intensity of the (100) peak is extremely high, suggesting that the (100) planes are probably the major growth direction. Figure S5c confirmed the KV_3_O_8_ had nanofibers feature with a length of 10–20 μm and diameter of around 100 nm. As shown in Figure S5d, the monoclinic LiV_3_O_8_ (JCPDS 35-0437, space group: P21/m) had typical diameters of around 200 nm and the nanowire morphology.


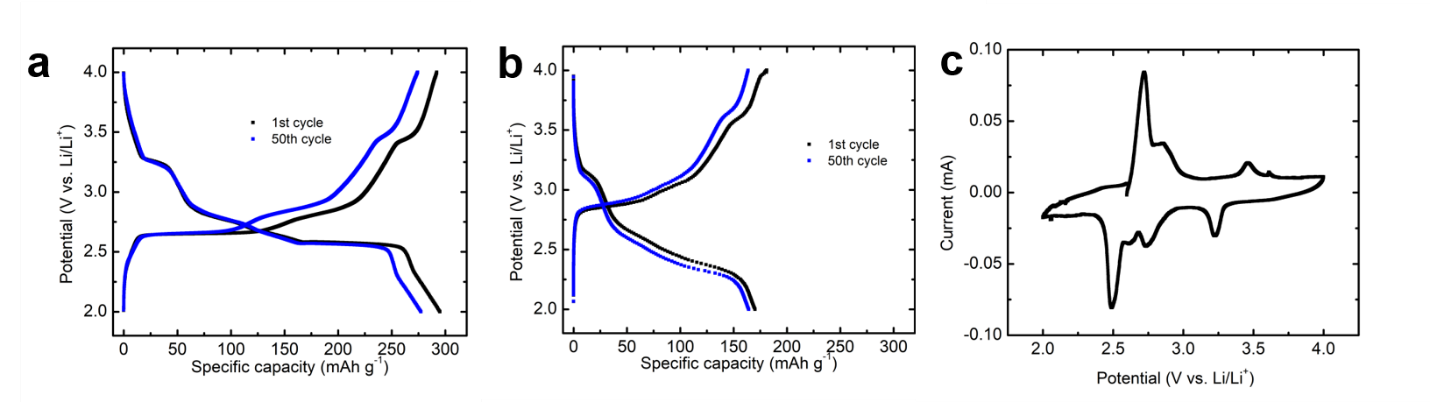


**Figure S5:** LiV_3_O_8_ in Li-ion battery, with Li metal as counter and reference electrode and 1 M LiPF_6_ in EC:DMC as electrolyte. First and 50th cycle galvanostatic charge-discharge cycles tests achieved at (a) 100 mA g^-1^ and (b) 2 A g^-1^; (c) CV curve test at 0.1 mV s^-1^. The above electrochemical performance of LiV_3_O_8_ synthesized by the molten salt method confirms that the molten salt method offers interesting opportunities for synthesizing nanomaterials with excellent electrochemical performance.


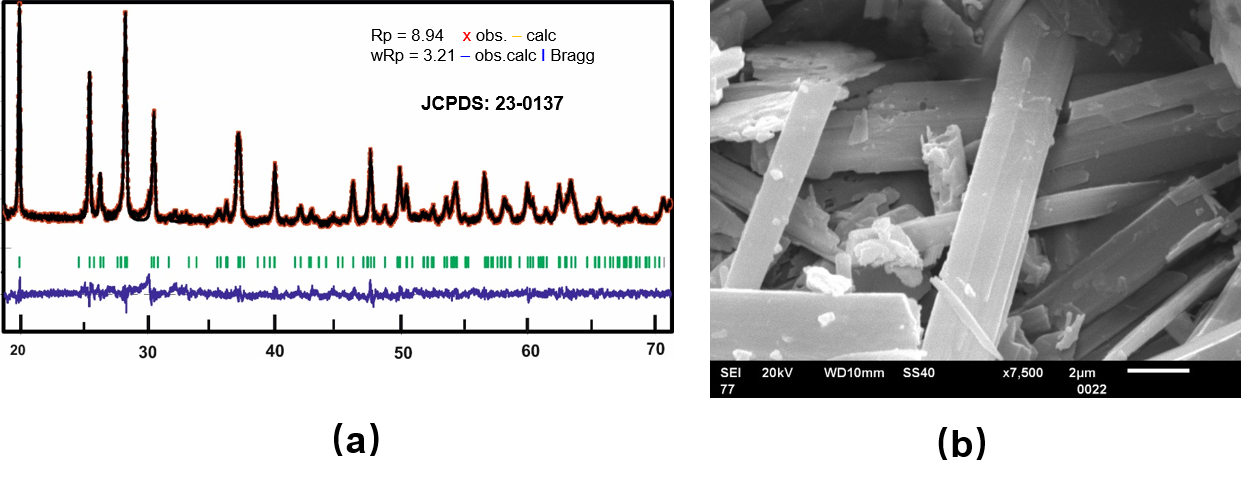


**Figure S6:** Characterization of CaV_2_O_6_ (a) X-ray diffraction pattern, (b) SEM image.


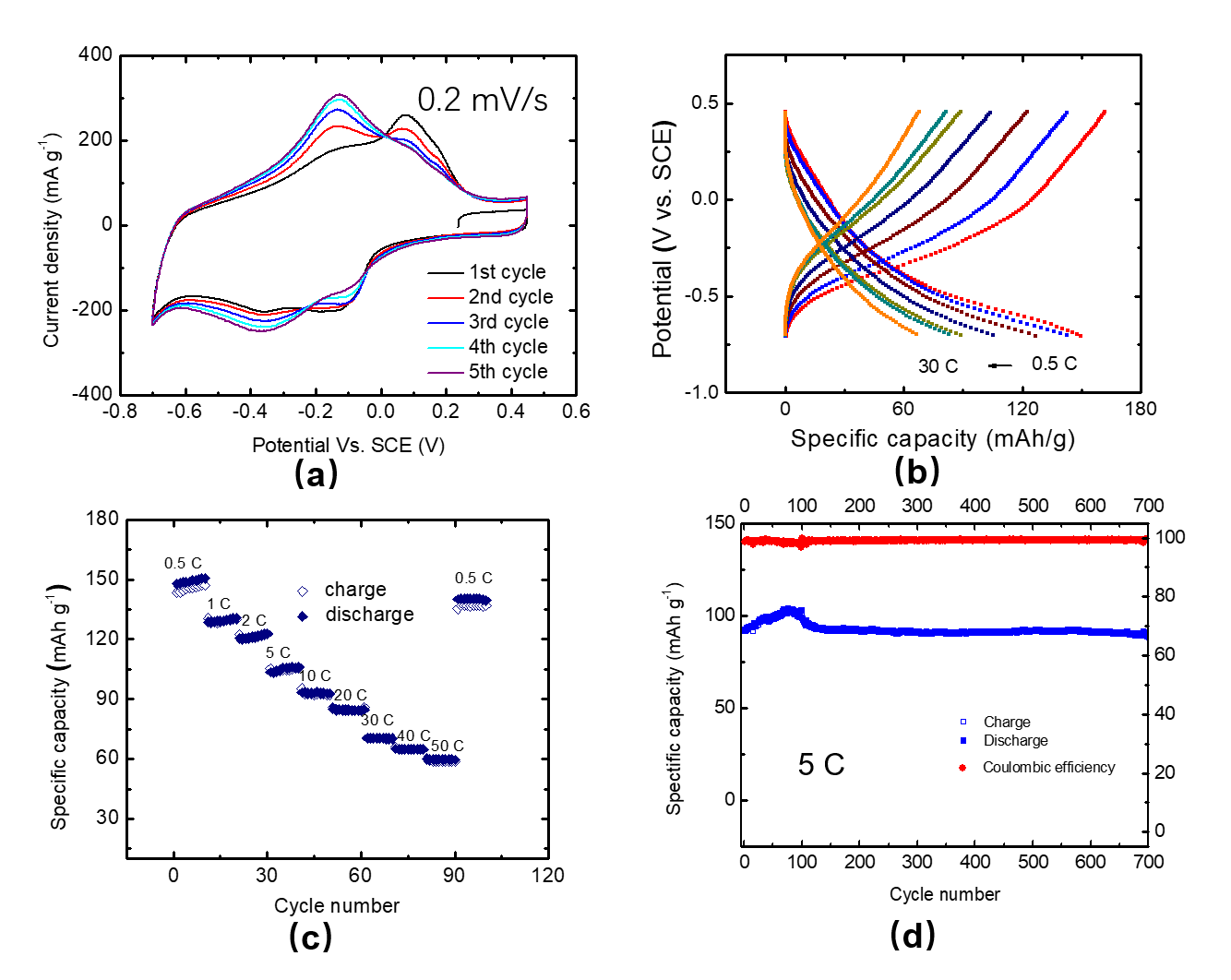


**Figure S7:** Electrochemical performance of CaV_2_O_6_ (a) Initial five first CV cycles of recorded at 0.2 mV s^-1^ (b) Galvanostatic charge-discharge profiles at different current densities. (c) Rate capability at varying C rates. (d) Cycling performance at a current density of 5 C.


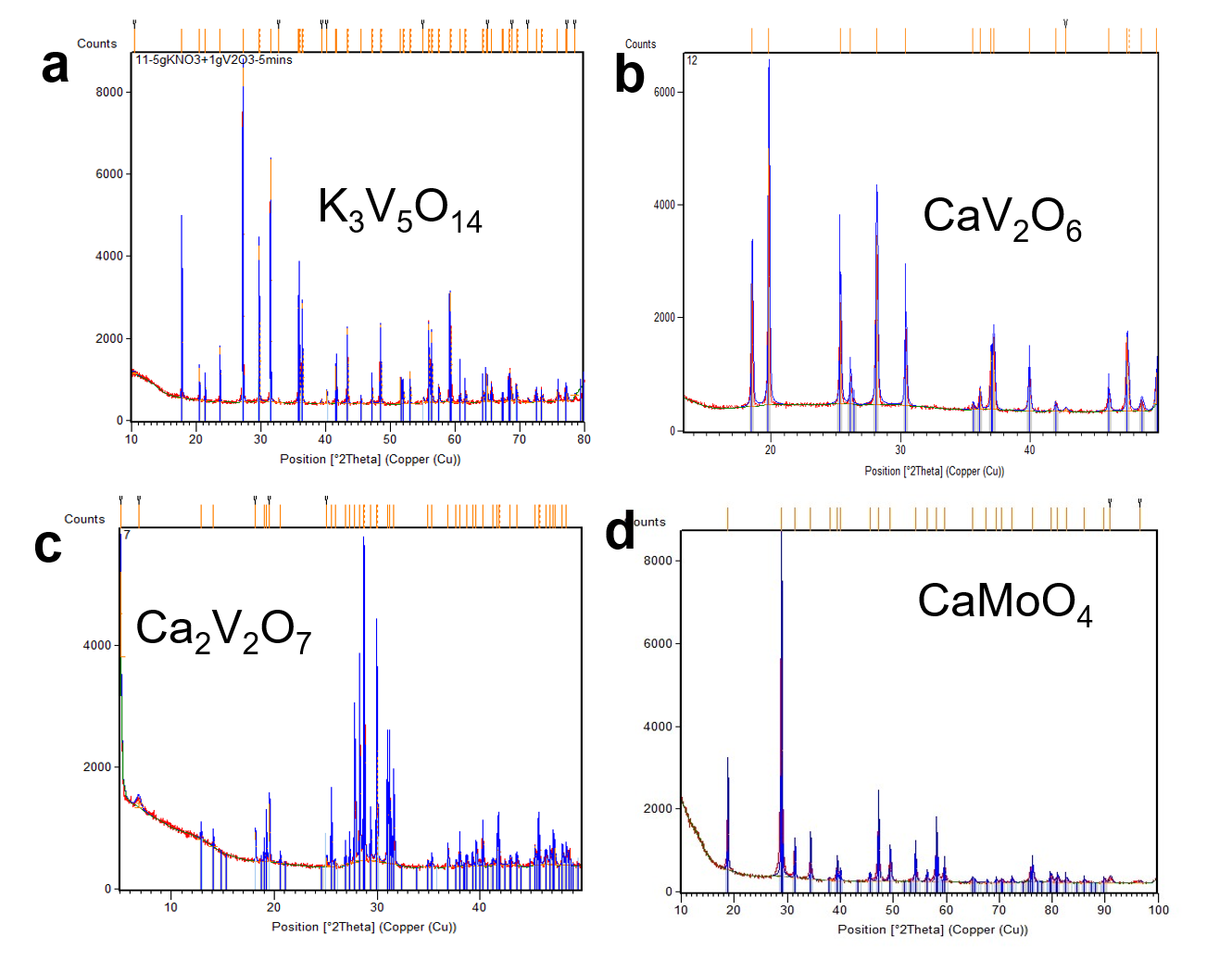


**Figure S8:** X-ray diffraction pattern of (a) K_3_V_5_O_14_, (b) CaV_2_O_6_, (c) Ca_2_V_2_O_7_ and (d) CaMoO_4_.

***
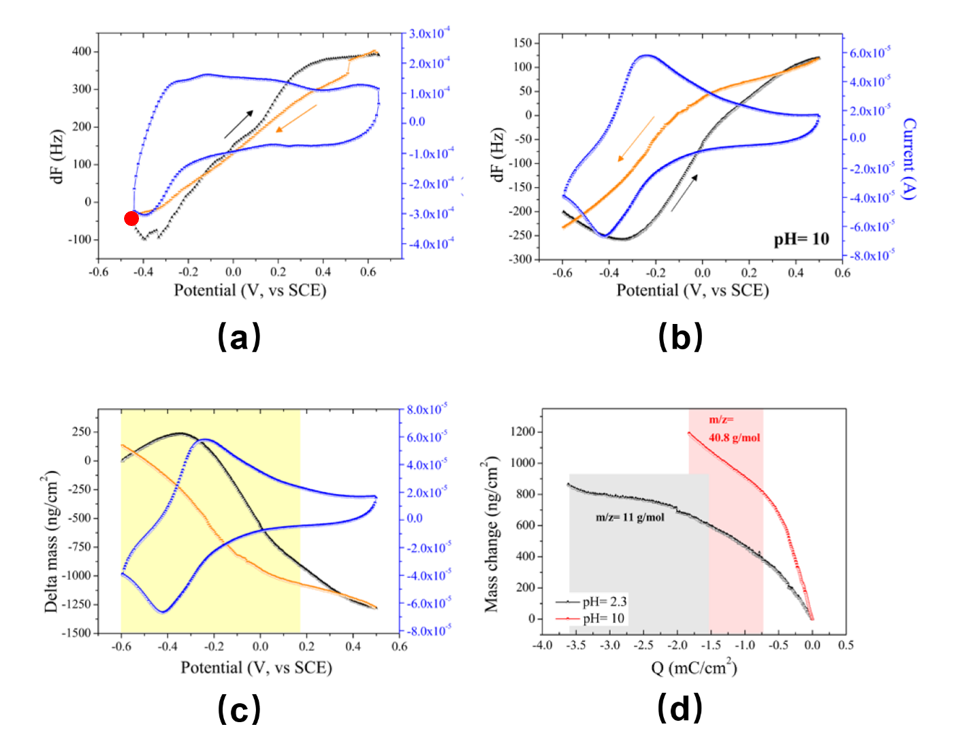
***

**Figure S9: CV and EQCM frequency response (a)** in pH= 2.3 aqueous electrolyte at 20 mV s^-1^. **(b)** in pH=10 aqueous electrolyte at 10 mV s^-1^. **(c)** CV of CVO in pH= 10 aqueous electrolyte and the corresponding mass change at 10 mV s^-1^. **(d)** Electrode mass change vs charge during the polarization of CVO in pH=2.3 (4.5M Ca(NO_3_)_2_) and pH=10 (4.5M Ca(NO_3_)_2_ + Ca(OH)_2_) electrolytes at 10 mV s^-1^.

EQCM study was achieved using the CVO electrode in the two electrolytes (pH =2.3 and pH=10), to get further information on the charge storage mechanism. To understand how the change of pH influences the charging mechanisms, EQCM has been performed. Figure S9a and S9b (blue lines) show the CVs of CaV_6_O_16_∙7H_2_O in two aqueous electrolytes with different pH, while the black and orange marks correspond to the associated frequency response measured by EQCM during the positive and the negative sweep, respectively. Both figures showed a frequency raise from the negative to the positive potential (black lines) and the other way around for the negative sweep (orange lines).

Figure S9c shows the electrode mass change during polarization in pH=10 aqueous electrolyte, calculated from Sauerbrey’s equation [5]. During the positive sweep corresponding to the oxidation of CVO, the mass decreased since ions deintercalated into the electrolyte (black line). Note that the weight increase at the low potential is due to the cathodic current upon potential reversal. On the other hand, from the positive to the negative potential, ion intercalation into CaV_6_O_16_∙7H_2_O results in an increase of mass (orange line). Figure S9d summarizes the results obtained in the two electrolytes, by plotting the change of the electrode weight versus the charge passed in the electrode during the reduction process (negative charges). Shaded areas represent the region of potential where the redox reaction occurs (the yellow zone for the pH=10 electrolyte), in which the average molecular weight of the ions intercalating was calculated. Average molar weight of m/z = 40 g∙mol^-1^ and 11 g∙mol^-1^ were obtained in the pH=10 and pH=2.3 electrolytes, respectively. As a result, the heavier molar weight obtained for pH=10 electrolyte would support the intercalation of heavier Ca^2+^ ion with one water molecule, while the intercalation of hydronium ions would explain the charge storage mechanism in acidic electrolyte.

Based on EQCM results, additional information can be obtained. From Figure S9a, the red dot represents the starting point of the selected cycle. For the sample in acidic (pH=2.3) aqueous electrolytes, the starting point overlaps the ending point which corresponds to the absence of irreversible mass loss during cycling. As a result, we exclude the possibility of the dissolution of the CVO electrode as a cause of the lower capacity in acidic (pH=2.3) aqueous electrolyte.


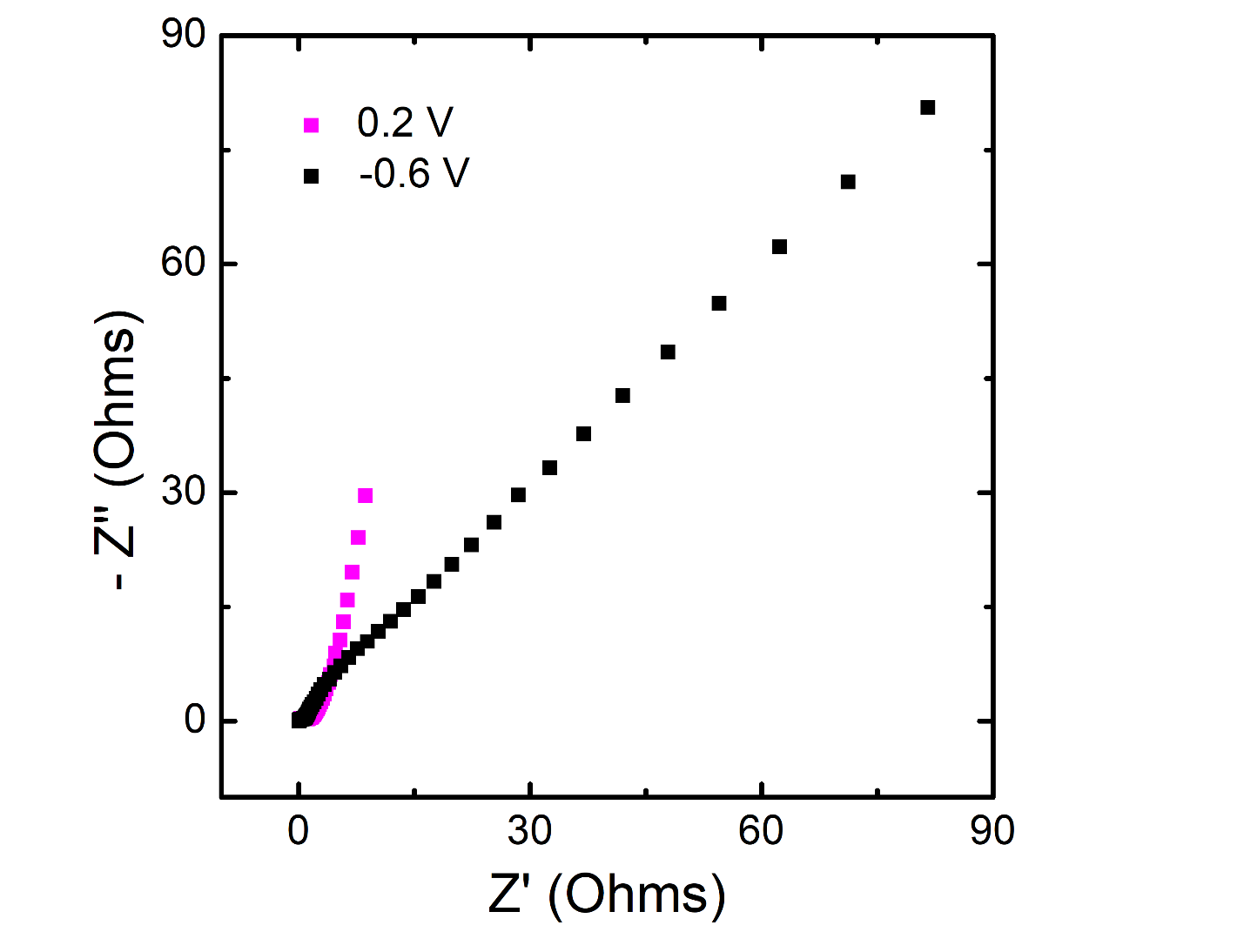


**Figure S10:** EIS analysis at different potentials at the 3rd charge (oxidation) cycle.

# References

[1] Lee, ChangHee, and Soon-Ki Jeong. “A Novel Superconcentrated Aqueous Electrolyte to Improve the Electrochemical Performance of Calcium-Ion Batteries.” Chemistry Letters 45, no. 12 (2016): 1447–49.

[2] J. Hyoung, J.W. Heo, S.T. Hong, Investigation of electrochemical calcium-ion energy storage mechanism in potassium birnessite, J. Power Sources. 390 (2018) 127–133.

[3] Wang, Richard Y., Colin D. Wessells, Robert A. Huggins, and Yi Cui. “Highly Reversible Open Framework Nanoscale Electrodes for Divalent Ion Batteries.” Nano Letters 13, no. 11 (2013): 5748–52.

[4] S. Gheytani, Y. Liang, F. Wu, Y. Jing, H. Dong, K.K. Rao, X. Chi, F. Fang, Y. Yao, An Aqueous Ca-Ion Battery, Adv. Sci. 4 (2017) 1–7.

[5] G. Sauerbrey, Verwendung von Schwingquarzen zur Wägung dünner Schichten und zur Mikrowägung, Zeitschrift Für Phys. 155 (1959) 206–222.
